# Endothelial Progenitor Cells inhibit jaw osteonecrosis in a rat model: A major adverse effect of bisphosphonate therapy

**DOI:** 10.1038/s41598-019-55383-5

**Published:** 2019-12-11

**Authors:** Tal Tamari, Rina Elimelech, Gal Cohen, Talia Cohen, Ofri Doppelt, Lana Eskander-Hashoul, Hadar Zigdon-Giladi

**Affiliations:** 1 Department of Periodontology, School of Graduate Dentistry, Rambam Health Care Campus, Haifa, Israel; 2 Laboratory for Bone Repair, Rambam Health Care Campus, Haifa, Israel; 3 The Ruth and Bruce Rappaport Faculty of Medicine, Technion - Israel Institute of Technology, Haifa, Israel

**Keywords:** Cell delivery, Translational research

## Abstract

Medication-related osteonecrosis of the jaw (MRONJ) is a serious adverse effect of antiresorptive and antiangiogenic therapies. MRONJ is identified by chronic wounds in the oral mucosa associated with exposed necrotic bone. We hypothesized that zoledronic acid (ZOL) impairs keratinocyte and fibroblast function and reduces soft tissue vascularization; therefore, treating MRONJ with proangiogenic cells may benefit MRONJ patients. The effect of ZOL and dexamethasone (DEX) on gingival fibroblasts and keratinocytes was investigated. *In-vitro*, ZOL inhibited fibroblast and keratinocyte proliferation, delaying scratch healing. *In-vivo*, exposed bone was detected at tooth extraction sites, mainly in ZOL(+)/DEX(+) rats; and was associated with significantly decreased soft tissue vascularization, serum-VEGF, and tissue-VEGF. Local injection of early and late endothelial progenitor cells (EPCs) healed 13 of 14 MRONJ lesions compared with 2/7 lesions in the mesenchymal stem cells, and 2/6, in culture-medium group. The EPCs reduced necrotic bone area, increased serum and tissue VEGF levels. EPCs engraftment was minimal, suggesting their paracrine role in MRONJ healing. The EPC-conditioned medium improved scratch healing of keratinocytes and fibroblasts via VEGF pathway and elevated mRNA of VEGFA and collagen1A1. In conclusion, a novel MRONJ treatment with EPCs, increased vascularization and improved epithelial and fibroblast functions as well as cured the lesion.

## Introduction

Medication-related osteonecrosis of the jaw (MRONJ) is a serious adverse event associated with pharmaceutical agents used to treat osteoporosis, Paget’s disease, hypercalcemia, multiple myeloma and metastasis in oncology patients. Treatments include bisphosphonates (BP), RANK ligand inhibitors, or antiangiogenic therapies^1^. The clinical manifestation of MRONJ includes the presence of exposed bone in the oral cavity for more than 8 weeks in a patient with previous or current use of one or more of these medications, with no history of radiation therapy.

The pathophysiology of MRONJ has not been fully elucidated. However, several known risk factors were reported such as age, periodontal disease, smoking, diabetes, steroid therapy, and immunosuppression^1^. The main theory for MRONJ pathogenesis is associated with interrupted bone remodeling due to impaired osteoclast function^1^. Recent evidence of MRONJ in patients taking anti-angiogenic drugs raises the possibility that an additional mechanism such as impaired soft tissue healing may cause the disease. Adequate soft tissue healing^2–5^ requires coordination between epithelial and fibroblast function, immune response, and adequate vascularity^6^. Previous studies noted that angiogenesis suppression can lead to MRONJ development, and that serum VEGF levels might be a predictive marker for it^7^. Similarly, cancer patients treated with zoledronic acid (ZOL) exhibited decreased serum VEGF^8^. The cytotoxic effect of ZOL on cultured fibroblasts and keratinocytes has also been previously published^9–11^.

Gingival fibroblasts play a critical role in the proliferative phase of oral wound healing^12^ by secreting extra-cellular matrix proteins and angiogenic growth factors, including VEGFA, to generate new tissue^4,5^. To date no standardized MRONJ prevention or treatment strategies exist. In order to investigate MRONJ pathogenesis, several research groups established rat models for MRONJ using systemic injection of bisphosphonates^13,14^. Sonis *et al*. described a MRONJ rat model using subcutaneous injection of zoledronic acid (ZOL) and dexamethasone (DEX) coupled with tooth extraction^14^. In this model, MRONJ-like lesions were replicating clinical, radiographic, and histologic features described in humans. In this study we investigated a cell-based therapy that utilized endothelial progenitor cells (EPCs) as a treatment modality for MRONJ in a rat model. Circulating EPCs participate in neovascularization^15,16^. Preclinical studies found that EPCs stimulate bone healing by increasing local angiogenesis^17,18^.

We hypothesize that zoledronic acid (ZOL) and dexamethasone (DEX) interrupt normal soft tissue wound healing and exacerbate MRONJ. Our goal was to follow the effect of ZOL and DEX on the *in-vitro* wound healing of keratinocytes and fibroblasts, and to evaluate vascular alterations in the oral mucosa in a MRONJ rat model. Furthermore, we investigated the potential for human EPCs to cure MRONJ based on this model.

## Materials and Methods

There were two major parts to this study: (1) *In-vitro* studies that included proliferation and scratch wound assays for human keratinocytes and gingival fibroblasts. (2) *In-vivo* studies that involved transplantation of human stem/progenitor cells in a rat model.

All experiments were performed in accordance with the Helsinki committee for human experiments, Rambam Health Care Campus (Helsinki number 0397-12 RMB) and the Committee for the Supervision of Animal Experiments at the Technion’s Ruth and Bruce Rappaport Faculty of Medicine (approval # IL0580514). All methods were performed in accordance with the relevant guidelines and regulations.

### *In-vitro* studies

All the experiments were repeated three times in triplicate.

#### Gingival fibroblasts and keratinocyte cell proliferation

Human primary gingival fibroblasts (GF) (ATCC PCS-201-018, Manassas, VA, USA) were cultured in Dulbecco’s modified Eagle medium (DMEM, BI, Beit-Haemek, Israel) high glucose, 10% FBS (BI, Beit-Haemek, Israel). Adult human keratinocytes (HaCaT) were cultured as described previously^19^.

To evaluate the effect of ZOL and DEX on fibroblast and keratinocyte function, 5 × 10^3^ cells were seeded in a 96-well plate and cultured with DMEM for 24 h. Zoledronic acid (Actavis Italy, Milan), DEX (Kern-pharma, Barcelona, Spain), ZOL + DEX (10 µM) were then added to the medium and incubated for an additional 72 h. To determine the effective concentration of the drugs a dose response assay was performed, see online Supplementary Fig S1. XTT assay (BI, Beit-Haemek, Israel) was performed according to the manufacturer instructions at 24, 48 and 72 h. Results were analyzed with an Elisa plate reader.

#### Scratch wound assay

Graduated 96-well plates from ESSEN were used to seed 2 × 10^4^ GF or 2.5 × 10^4^ keratinocytes. When cells reached 95% confluence, a wound was made on every well using Wound Maker 96 (Essen BioScience, MI, USA). Cell migration toward the wounds was monitored every two hours and analyzed by the ESSEN IncuCyte system. Determination of the effective drug concentration was performed and presented online in Supplementary Fig S2.

#### Isolation, expansion and characterization of early and late EPCs

Human EPCs were isolated from the blood of two healthy volunteers (Helsinki number 0397-12 RMB) in accordance with the Good Clinical Practice (GCP) guidelines and regulations and informed consent was obtained. For cell isolation 50 mL blood was obtained from healthy volunteers who signed an informed consent. Pooled peripheral blood was collected into sterile heparinized tubes. Blood was diluted 1:1 with phosphate-buffered saline (PBS). Mononuclear cells (MNCs) were isolated with density gradient centrifugation (Lymphoprep, Axis-Shield) and pelleted cells were resuspended in Endothelial Basal Medium (EBM-2) containing 20% fetal bovine serum (FBS), penicillin-streptomycin (Biological Industries Ltd.) and supplemented with Endothelial Growth Medium (EGM-2MV SingleQuote; Clonetics, Cambrex Bio Science) that includes: vascular endothelial growth factor, fibroblast growth factor-2, epidermal growth factor, insulin-like growth factor-1 and ascorbic acid. Cells were seeded on six-well plates coated with 5 µg/cm^2^ of fibronectin (Biological Industries Ltd.) and grown at 37 °C with humidified 95% air/5% CO _2_. After 4 days of culture, nonadherent cells were discarded by gentle washing with PBS, and fresh medium was applied. The attached cells were continuously cultured with complete EGM-2 medium. Ten days after Isolation, early EPCs were characterized. 14–21 days after isolation, late EPCs were identified in the culture plate by the pretense proliferative cells colonies. Cells were fed three times per week and were split when they reached ~80% confluent by brief trypsinization using 0.5% trypsine in 0.2% ethylenediaminetetraacetic acid (EDTA; BI, Beit-Haemek, Israel.). EPC were characterized using flow cytometry (fluorescence-activated cell sorting, FACS) using fluorescein isothiocyanate-labeled antibodies specific for: CD14, CD34 (mouse anti-human, BD Biosciences, San jose, CA, USA) and CD31 (LifeSpan BioSciences, Seattle, Washington, USA), KDR (mouse anti-human, BD Biosciences). In this study, 5 × 10^5^ cells in PBS were incubated 30 min with antibodies according to the manufacturers’ recommendations. Negative controls were mouse immunoglobulin (Ig)G1 isotype (BD Biosciences). Following washings ×3, cells were resuspended in PBS and analyzed using FACScan and CellQuest software (Becton Dickinson & Co, San jose, CA, USA). Early EPCs expressed CD31 (98%), CD 34 (94%), CD 45 (<0%), CD 14 (98%) and KDR (<0%). Late EPCs expressed CD31 (98%), CD 34 (94%), CD 45 (2.7%), CD 14 (5.1%) and KDR (48%).

#### EPC Conditioned medium (EPC-CM) preparation and VEGF measurements

One million human EPCs were cultured in EGM-2 until 80% confluence. After incubation for 48 h, 10 ml medium was collected and concentrated using a centrifugal filter (Merck Millipore, Tullagreen Ireland). Concentrations of VEGF in the EPC-CM were analyzed using ELISA kit (R&D Systems, MN, USA) according to the manufacture protocol.

#### VEGF neutralization and VEGF pathway blocking

Neutralization of VEGF in EPC-CM was performed by VEGF receptor 2 (VEGFR-2) antibody (R&D Systems, MN, USA). 0.2 mg/ml VEGFR-2 antibody was added to the EPC-CM at room temperature. Furthermore, VEGF pathway was blocked using phosphoinositide 3-kinases inhibitors (PI3K, LY-294,002, Sigma-Aldrich, MS, USA) that was added to EPC-CM in two concentrations: 50 µM and 10 µM.

#### Expression of VEGFA and collagen1A1 genes

To evaluate the effect of EPC-CM on GF and keratinocytes, 2 × 10^5^ cells were cultured for 30 h with DMEM + 10 µM ZOL; after 48 h the medium was replaced with fresh drug-free medium supplemented with EPC-CM, endothelial growth medium without EPC secretome (EGM-2), and DMEM. RNA was extracted from cell pellets with the RNeasy mini kit (Qiagen, Hilden, Germany) using the Qiacube automated system (Qiagen, Hilden, Germany). 1 µg RNA from each sample was taken to RT reaction. cDNA was generated with High-Capacity cDNA Reverse Transcription Kit (ThermoFisher Scientific, Massachusetts, USA). Quantitative real-time PCR analysis (qPCR) was performed using real time PCR (Biometra Analytik, Jena, Germany) and syber green (Fast SYBR™ Green Master Mix, Applied Biosystems™, California, USA) All primers were supplied by Syntezza Bioscience (Jerusalem, Israel) VEGFA forward: TCTTCAAGCCATCCTGTGTG reverse: TGCATTCACATTTGTTGTGC and COL1A1 forward: GAGCGTGGTGTGCAAGGT, reverse: ACCCTTAGGCCCTGGAAGAC. The normalizing gene was HPRT1 forward: HPRTGACCAGTCAACAGGGGACAT, reverse: CCTGACCAAGGAAAGCAAAG. Data were analyzed with the relative Quantification-comparative CT method.

### *In-vivo* rat model

The study protocol was approved by the Committee for the Supervision of Animal Experiments at the Technion’s Ruth and Bruce Rappaport Faculty of Medicine (approval # IL0580514). All experiments were performed in accordance to ARRIVE guidelines and regulations.

#### Defining the model in rats

The model was defined using male and female Lewis inbred rats (*n = *50, 13 weeks, ~300 g), which were treated according to a previously published protocol by Sonis *et al*.^14^ with a subcutaneous injection of one of the following, once a week for 11 weeks: ZOL 7.5 µg/Kg (n = 9); DEX 1 mg/kg (n = 9); ZOL 7.5 µg/Kg with DEX 1 mg/kg (ZOL(+)/DEX(+)) (n = 21); (control): saline (n = 11)^20^. On week three, rats were anaesthetized by intramuscular injection of 100 mg/kg bw Ketamin (Ketaset, Fort Dodge, Iowa, USA) and 5 mg/kg bw Xylazin (Eurovet, Cuijk, Holland) and underwent bilateral first maxillary molar extraction. Three days post extraction, rats were treated with subcutaneous injection of 0.3 mg/kg Buprenorphine (Vetamarket, Shoham, Israel) and 50 mg/kg Cephalexin (Norbrook laboratories, Newry, Ireland) that were injected s.c. Rats were kept in ventilated cages (2–3 rats in each cage) and fed chow softened in water and water ad libitum. ZOL and DEX administration was maintained until sacrificed, 8 weeks after teeth extraction.

#### Treatment of MRONJ-like lesion using stem/progenitor cells

Male and female (*n* = 50) Lewis inbred rats were treated with ZOL and DEX as described above. Eight weeks after tooth extraction, macroscopic intra-oral examination was performed to detect exposed bone or fistula, and photographed. Rats demonstrating normal healing were sacrificed (*n* = 24) while rats that showing MRONJ signs were randomly divided into four treatment groups: (1) standard medium (endothelial basal medium (Lonza, Basel, Switzerland) or DMEM without serum or supplements (n = 6); (2) early EPC (n = 6); (3) late EPCs (n = 7); 4) mesenchymal stem cells (MSC; n = 7).

Stem cell preparation and injection: Human EPCs were isolated from the blood of two healthy volunteers (Helsinki number 0397-12 RMB), as described above^21^. Bone marrow human MSCs were cultured as previously described^22^. Before transplantation, hEPCs and hMSCs were suspended in serum free medium. Finally, 5 × 10^5^ cells suspended in 150 µL were injected into the gingiva around the lesion.

Serum VEGF analysis: Before sacrifice, 1 ml blood was collected from the heart and centrifuged. Serum-VEGF was analyzed using ELISA kit (R&D Systems, MN, USA) according to manufacturer instructions.

3D visualization and quantification of functional vessels via lightsheet microscopy: Before sacrifice, Dil solution (Thermo Fischer, MA, USA) was perfused into the internal carotid artery.

Dil molecules are directly incorporated into the membrane of cells thereby label functional vessels in red. Dil solution was prepared immediately before injection by diluting 200 µL of Dil stock solution in 10 ml of 5% glucose solution. A ten centimeter para-median incision was made along the neck and the internal carotid artery was isolated and ligated (silk 6-0 suture). An 20 g IV cannula (Terumo medical corporation, NJ, USA) was inserted into the artery and 3 ml Dil solution was perfused (1 ml per minute). Then, animals were sacrificed with 1 ml Pentobarbital sodium (CTS Chemical Industries, Kiryat Malachi, Israel) injection to the heart and 1 ml paraformaldehyde (PFA) 4% (Sigma-Aldrich, MS, USA) was perfused via the cannula. A sample of the oral mucosa (1 × 2 mm) was excised from the extraction healing site and embedded in 1% agarose gel. 3D visualization of functional vessels was performed using a Lightsheet Z.1 microscope (Zeiss, Oberkochen, Germany).

Clinical Evaluation of MRONJ occurrence: Eight weeks after extraction, mucosal healing or exposed bone were photographed. The MRONJ lesions treated with culture medium, stem, or progenitor cells were photographed before and four weeks after treatment.

Histology and histomorphometry: Paraffin sections were stained with hematoxylin and eosin. The extraction site area was identified adjacent to the second molar and defined as the area of interest for histomorphometric analyses. Empty lacunae were counted manually and necrotic bone was defined when the number of empty lacunae was greater than three^23^. The area of necrotic bone was measured using Image-Pro premier software (Rockville, MD, USA).

Immunohistochemistry: Histological slides were immuno-stained with rabbit anti rat CD31 (1:100, Novus Biologicals, Littleton, USA); rabbit anti-rat VEGF (1:400, Novus Biologicals, Littleton, CO, USA) and HNA (1:100, Scytek, Utah, USA). Hematoxylin stain was used for general morphology. Quantitative analysis of immunohistochemistry results was performed automatically using image-Pro premier software (Rockville, MD, USA).

### Statistical analysis for all studies

SPSS v25 (IBM, NY, USA) statistical software was used. Differences between groups were tested by Kruskal Wallis test with adjustments for multiple comparisons. Comparisons between control and the other groups was performed using non-parametric Mann-Whitney U test. The significance level was set at 5%.

## Results

### ZOL delays *in-vitro* wound healing of gingival fibroblasts

**Figure 1:**
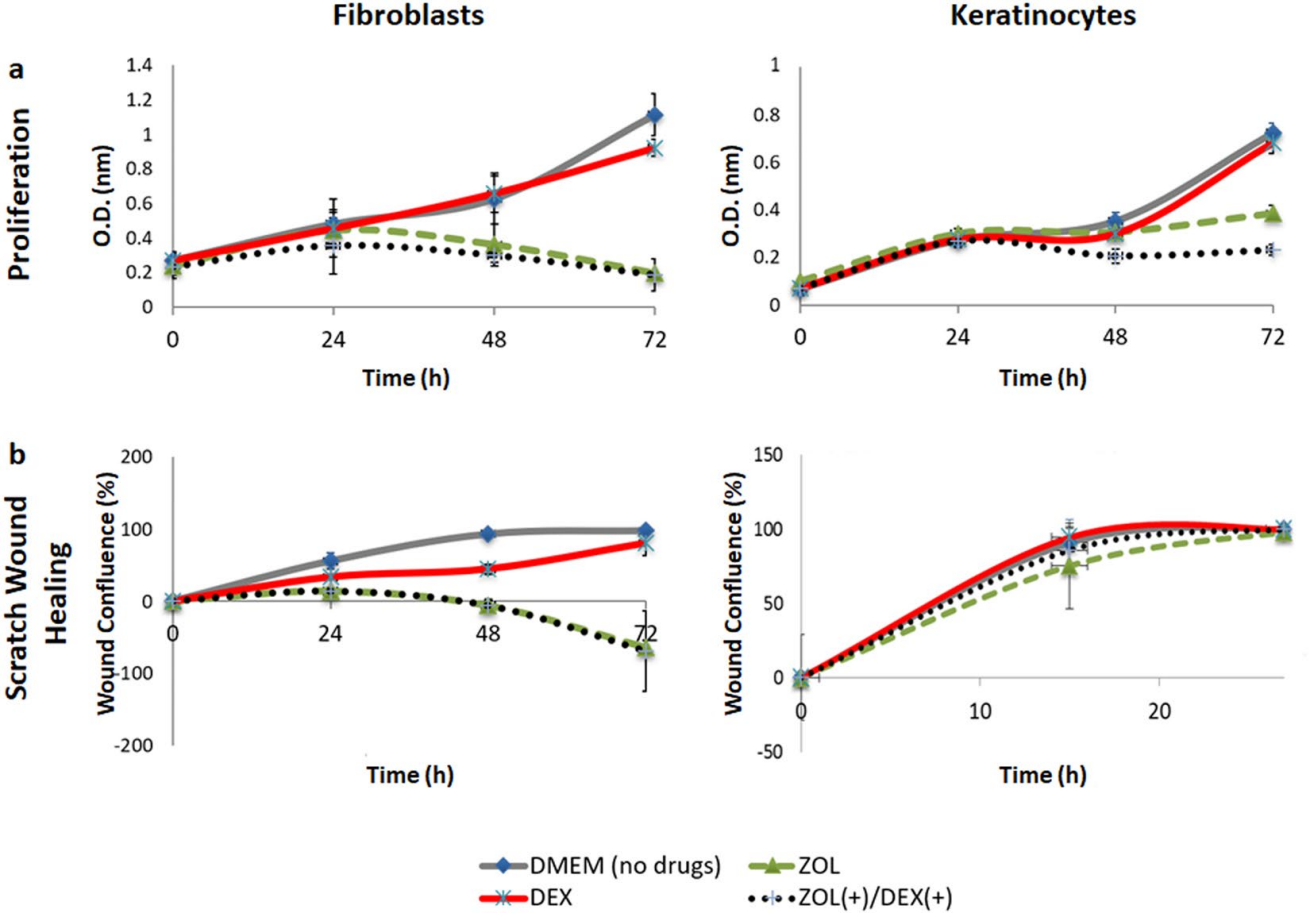
Effect of zoledronic acid (ZOL) and dexamethasone (DEX) on gingival fibroblast and keratinocyte functions. (**a)** Gingival fibroblasts and keratinocytes were cultured for 72 h with Dulbecco’s modified Eagle medium (DMEM) (control) or with 10 µM ZOL, DEX and ZOL(+)/DEX(+). Proliferation was analyzed with XTT. Cells cultured in 10 µM ZOL or 10 µM ZOL(+)/DEX(+) showed decreased viability after 48 and 72 h (*P* ≤ 0.03 *vs*. DMEM), whereas cells in the other groups demonstrated increased cell viability throughout the experiment. **(b)** Confluence of the wound was monitored every hour and analyzed using the IncuCyte software. GF treated with ZOL or ZOL(+)/DEX(+) showed delayed wound closure (*P* ≤ 0.004), whereas keratinocytes were less affected by the drugs. Experiments repeated tree times in triplicates.

The effect of ZOL and DEX on *in-vitro* wound healing was investigated in XTT and scratch wound assays using GF and keratinocytes. Generally, proliferation of primary adult human GF and adult human keratinocytes (HaCaT) treated with DEX were similar to cells cultured in standard medium. In contrast, treatment with 10 µM ZOL or 10 µM ZOL (+)/DEX(+) significantly reduced GF and keratinocyte proliferation at 48 and 72 h (GF at 48 and 72 h, *P* ≤ 0.0001 compared to DMEM; keratinocytes at 48 h *P* ≤ 0.03, at 72 h *P* ≤ 0.009 compared to DMEM; Fig. 1a). Similar GF trends were observed for the scratch wound healing assay: wound closure was slightly reduced by 10 µM DEX compared with medium. A more dramatic effect was found in the ZOL10 µM and ZOL (+)/DEX(+)10 µM groups, which demonstrated increase in the wound area at 48 h (p ≤ 0.004 ZOL, ZOL(+)/DEX(+) vs. DMEM) and 72 h (p ≤ 0.003 ZOL, ZOL(+)/DEX(+). No additive or synergistic effect of ZOL and DEX on GF proliferation and migration was detected, suggesting that 10 µM ZOL was responsible for the primary negative effect on the cells. Keratinocytes were less affected by the drugs and showed rapid wound closure (27 h) in all the treatment groups (Fig. 1b).

The effect of ZOL and DEX on mRNA levels of Receptor activator of nuclear factor kappa-Β ligand (RANKL) and Osteoprotegerin (OPG) was investigated. Small but significant reduction in RANKL expression was found in GF and keratinocytes following exposure to ZOL. Results can be found as Supplementary Table S1.

### Combined ZOL and DEX therapy increases incidence of chronic wound and MRONJ in a rat model

**Figure 2:**
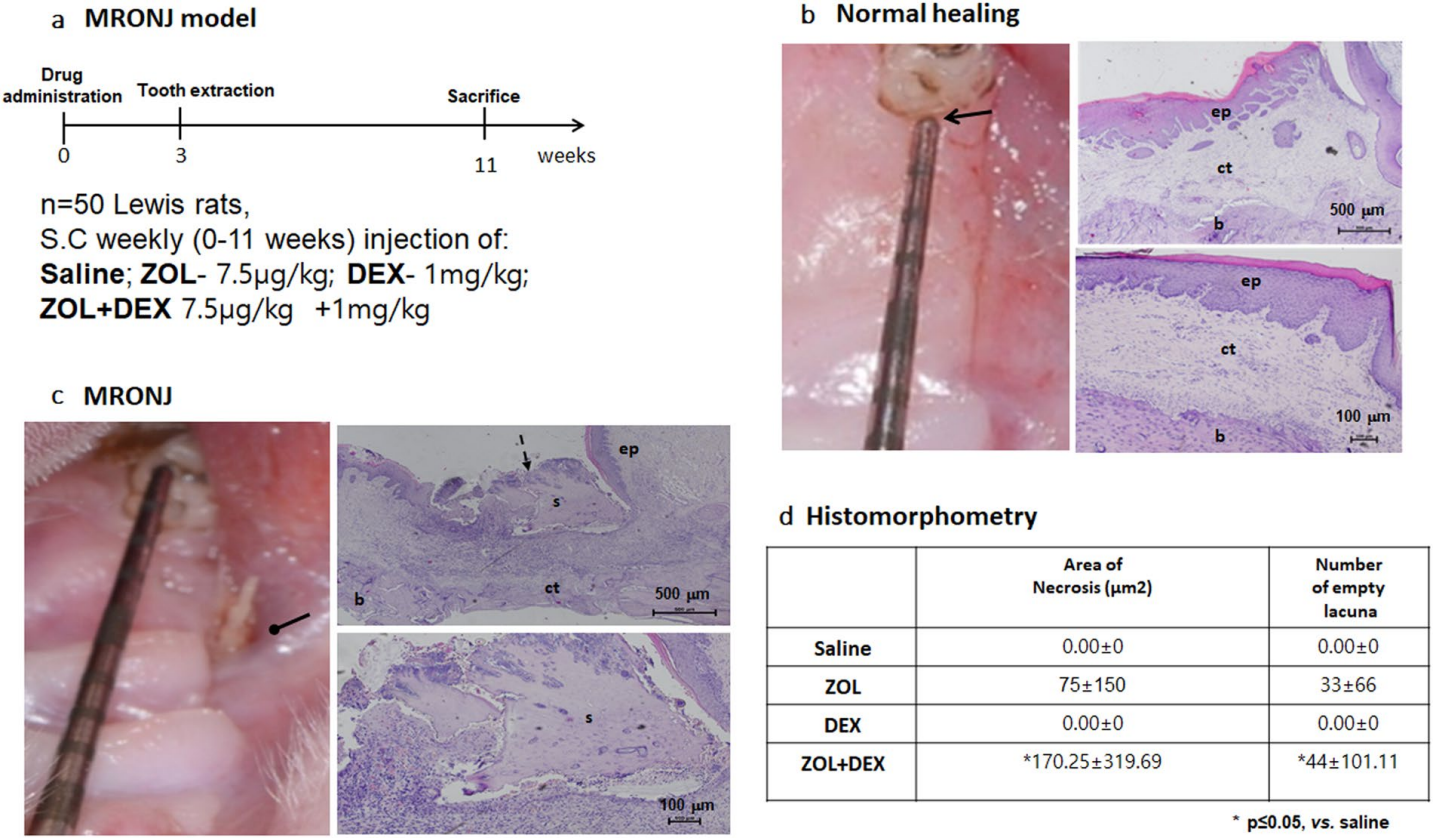
Effect of zoledronic acid (ZOL) and dexamethasone (DEX) on wound healing *in-vivo*. (**a)** Animal study flow chart. (**b**) Normal healing of extraction socket (arrow) was detected in all rats in saline (control) and DEX groups. Histological slides haematoxylin and eosin demonstrated intact epithelium (ep) and wide connective tissue (ct) covering vital bone (b) that filled the extraction socket. **(c)** Medication-related osteonecrosis of the jaw lesions, in the ZOL(+)/DEX(+) treated rats, characterized by the presence of exposed bone (elliptical arrow). Histological slides showed incomplete epithelium (ep) with areas of non-vital bone (sequestrum, s) extending from the epithelial opening (dashed arrows) and surrounded by inflammatory infiltrate. **(d)** Histomorphometric analysis of area of necrosis and number of empty lacunae at the extraction site. *P ≤ 0.05 ZOL(+)/DEX(+) *vs*. Saline.

*In-vitro* wound healing was mainly delayed by ZOL and ZOL(+)/DEX(+); therefore, the effect of ZOL, DEX and their combination was examined *in-vivo* (Fig. 2a)^24^. Rats were treated for 11 weeks with: ZOL, DEX, ZOL(+)/DEX(+) or saline. Rats underwent bilateral tooth extractions at week 3 and sacrifice at 11 weeks. Seven out of 50 rats died during anesthesia before or after tooth extraction. Animals that survived demonstrated normal behavior, and gained body weight. Clinically exposed bone or a fistula (MRONJ-like lesions) were evident in 50% of the ZOL(+)/DEX(+) group (20 out of 40 sites) and in 25% of the ZOL group (3 out of 12 sites; Fig. 2C). All saline (22 out of 22 sites) and DEX (12 out of 12 sites) treated rats presented complete mucosal coverage of the extraction sites (Fig. 2b).

Histological characteristics of MRONJ were found in rats with MRONJ-like clinical lesions. Rats in the saline and DEX groups demonstrated continuous epithelium, and wide non-inflamed connective tissue, with mature vital bone filling the tooth extraction socket. ZOL(+)/DEX(+) and ZOL rats that developed MRONJ-like lesions showed epithelium discontinuation with fragments of non-vital bone surrounded by non-specific inflammatory infiltrate (Fig. 2b,c). The total area of necrotic bone and number of empty lacunae were higher in the ZOL(+)/DEX(+) compared with saline (p ≤ 0.05). The ZOL, DEX groups were similar to the saline (p > 0.05) (Fig. 2d).

### ZOL, DEX, and their combination, reduce vascularity of the oral mucosa *in-vivo*

**Figure 3:**
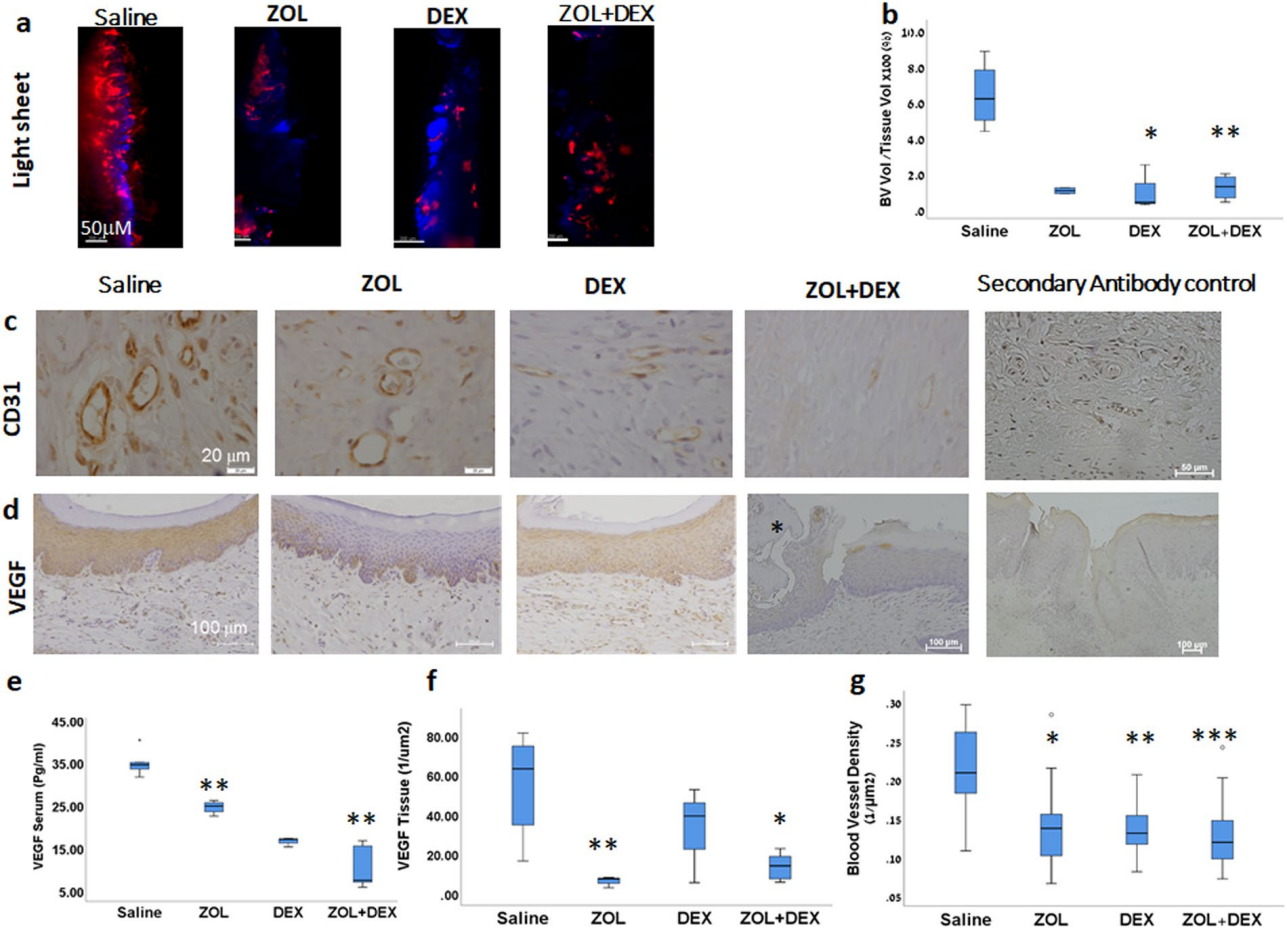
Decreased vascularity and vascular endothelial growth factor (VEGF) levels by zoledronic acid (ZOL) and dexamethasone (DEX), in a rat medication-related osteonecrosis of the jaw *(MRONJ) model*. (**a**) Functional blood vessels in the oral mucosa at the healing extraction site were labeled by Dil solution (red) and detected with Lightsheet Z.1 microscope cell nuclei stained in blue (Nuc Blue). (**b**) Quantitative analysis of blood vessel volume in the tissue. (**c**) Immunostaining of blood vessels in the oral mucosa using anti-rat CD31. (**d**) Immunostaining with anti-rat VEGF. Sequestrum was marked with asterisk (**e**) Serum VEGF levels measured by ELISA, 11 weeks following drug administration (**f**) Quantitative analysis of tissue-VEGF. (**g**) Quantitative analysis of blood vessel density *P < 0.05, **P ≤ 0.01, ***P ≤ 0.001, all groups vs. Saline.

Since reduced vascularity may lead to impaired wound healing, the effect of ZOL and DEX on soft tissue vascularization was investigated (Fig. 3). Serum-VEGF was measured 11 weeks after drug administration. Serum-VEGF was higher in the saline group (35.27 ± 3.23 pg/ml) and lowest in the ZOL(+)/DEX(+) group (10.75 ± 5.17 pg/ml, *P* = 0.009). Administration of ZOL (24.83 ± 1.54 pg/ml) or DEX (16.78 ± 1.08 pg/ml) also reduced serum VEGF compared with saline (35.27 ± 3.23 pg/ml) (*P* = 0.015 and *P* = 0.025, respectively; Fig. 3e).

Immunohistochemical staining with VEGF antibody was found mainly in the cytoplasm of oral keratinocytes. Highest levels were found in the saline group (56 ± 26 1/mm^2^) and significantly lower levels were measured in the ZOL (6.6 ± 2.8 1/mm^2^, *P* = 0.007) and ZOL(+)/DEX(+) groups (14.21 ± 6.8 1/mm^2^, *P* = 0.027). Reduces tissue-VEGF was also found in the DEX group (32.89 ± 24.27 1/mm^2^) as compared to the saline group (56 ± 26 1/mm^2^) (no statistical difference; Fig. 3d,f).

Oral mucosal blood vessel density was evaluated in 2D (using anti-CD31) and in 3D (following Dil perfusion). In both methods, ZOL(+)/DEX(+) treatment was associated with reduced vascularity of the oral mucosa. The volume of functional vessels in the tissue was lower in the ZOL(+)/DEX(+) (*P* = 0.007) and DEX groups (*P* = 0.021) compared to the saline group (Fig. 3a,b), and the number of blood vessel branches was lower in the ZOL(+)/DEX(+) group (*P* = 0.038, *vs*. Saline). Likewise, the number of CD31 luminal structures was significantly reduced in the ZOL, DEX, ZOL(+)/DEX(+) groups as compared to saline (*P* = 0.023, 0.004, 0.001, respectively; Fig. 3c,g).

### EPCs treatment stimulates healing of MROJ-like lesions

**Figure 4:**
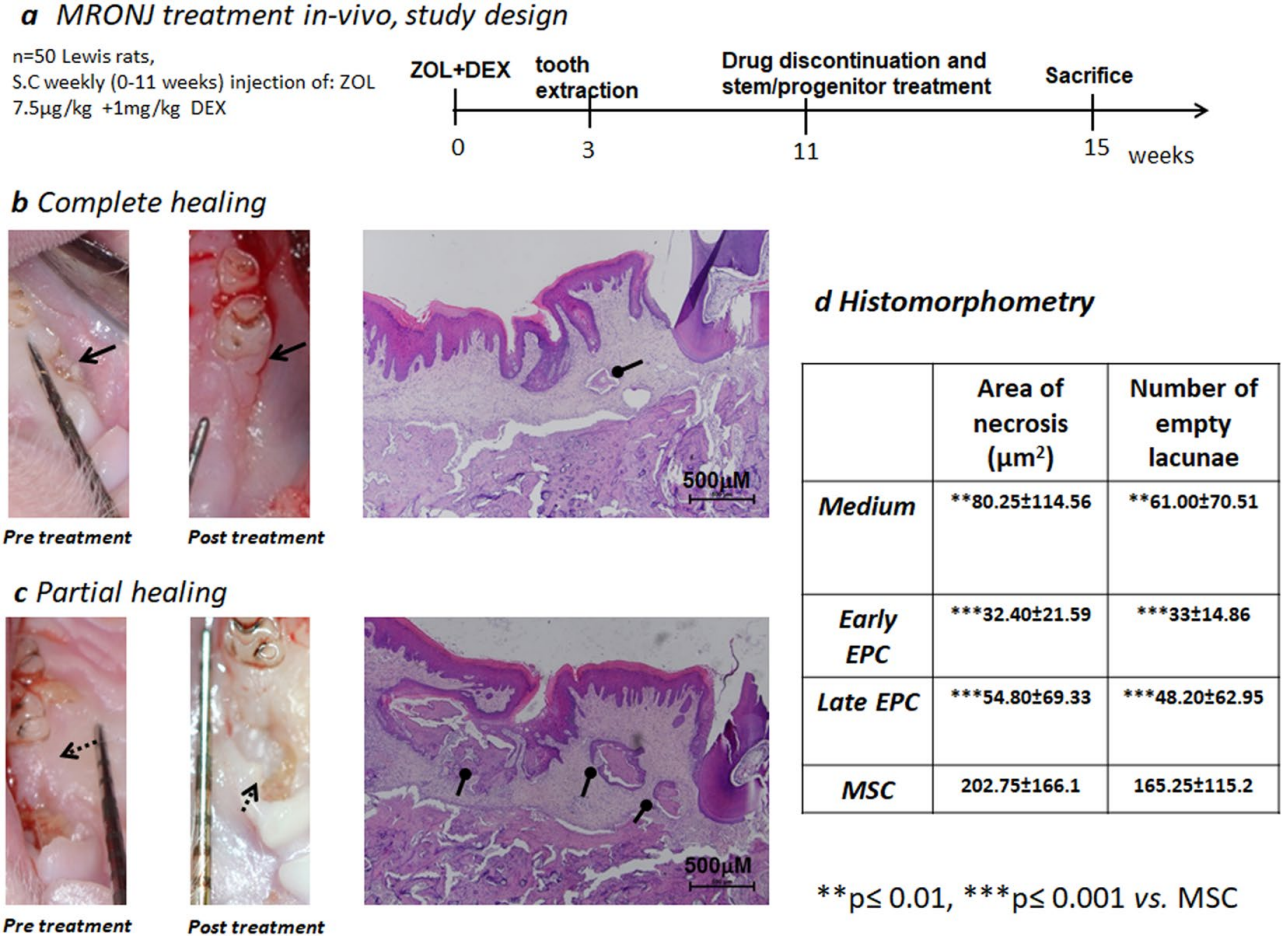
*Healing of* medication-related osteonecrosis of the jaw *(MRONJ) using stem/progenitor cells in a rat MRONJ model*. The clinical photographs with calibration scale were taken before and 4 weeks after treatment of MRONJ. **(a)** Animal study flow chart; (**b**) Representative images demonstrating complete healing of a 3 mm exposed bone (arrow) covered with soft tissue. Histological (haematoxylin and eosin staining) evaluation shows continuous oral mucosa and vital bone filling the extraction site. Small necrotic bone (elliptical arrow) was found (**c**) Representative images of incomplete healing showing partial coverage of the exposed bone (dashed arrows), with histological evidence of large areas of necrotic bone (elliptical arrows) at the extraction site. (**d**) Histomorphometric analysis of the necrotic area and number of empty lacunae at the extraction site, 4 weeks after treatment with stem/progenitor cells. ***P ≤ 0.0001, EPCs vs. MSCs, **P ≤ 0.001, Medium vs. MSC.

To improve vascularity, we suggested treating MRONJ with proangiogenic cells *in-vivo* (Fig. 4a). Following 11 weeks of treatment with ZOL(+)/DEX(+), exposed bone was photographed clinically, and the lesions were treated with a local subcutaneous injection of 5 × 10^5^ human primary cells: EPC, MSC, or cell growth medium (referred to hereinafter as “medium”). Four weeks after cell therapy, comparison of pre- with post-treatment photographs showed 12/13 early and late EPC-treated MRONJ-like lesions healed whereas only 2/7 lesions in the MSC, and 2/6 lesions in the medium group showed complete healing. MRONJ-like lesions treated with early or late EPCs showed superior healing histologically, compared with other treatment groups (Fig. 4b,c). The necrotic area and number of empty lacunae were lowest in the early and late EPCs, moderate in the medium, and highest in the MSCs groups (*P* ≤ 0 0.0001 early and late EPCs *vs*. MSC). Differences were also observed between the medium and MSC groups (*P* = 0.006; Fig. 4d).

### EPCs and MSCs elevate serum and tissue VEGF levels

**Figure 5:**
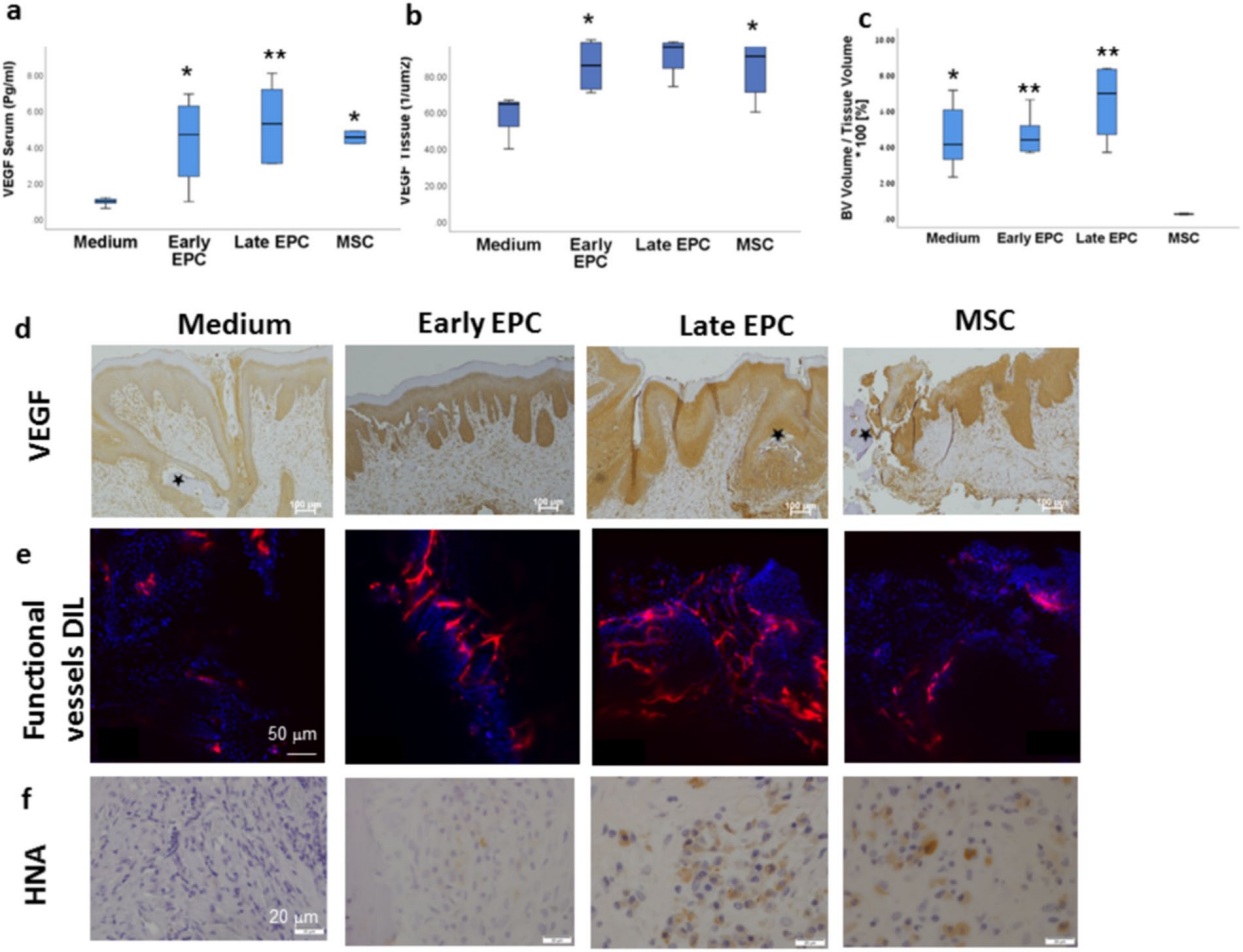
Increased serum and tissue vascular endothelial growth factor (VEGF) levels following medication-related osteonecrosis of the jaw (MRONJ) treatment with stem/progenitor cells. (**a**) Serum-VEGF levels measured by ELISA, 4 weeks following treatment. (**b**) Quantitative analysis of tissue-VEGF. (**c**) Quantitative analysis of blood vessel density. **P = 0.004, **P = 0.034, *P = 0.051 compared to MSC(**d**) Immunostaining with anti-rat VEGF. Sequestrum was marked with asterisks (**e**) Functional blood vessels were labeled with Dil solution. Florescent signal in the oral mucosa was detected with LSM 510 Zeiss laser confocal system (Zeiss, Germany). (**f**) immunostaining with HNA. *P ≤ 0.05, **P ≤ 0.001, compared to Medium.

Four weeks after cell therapy, blood was collected and serum VEGF measured. Lowest levels were observed in the medium group (0.97 ± 0.21 pg/ml). A fourfold increase in serum-VEGF was found in rats treated with all cell types (*P* = 0.05, *P* = 0.01, *P* = 0.02 early EPC, late EPC, and MSC compared to medium, respectively) (Fig. 5a). Cell therapy elevated VEGF in the oral keratinocytes (*P* = 0.05 early EPCs and MSCs compared with medium) (Fig. 5b,c). Blood vessel density was highest in the EPCs and lowest in the MSC groups (*P* = 0.004 Late EPCs vs. MSC; *P* = 0.034 early EPCs vs. MSC) (Fig. 5c,e). Immunostaining with HNA demonstrated the presence of transplanted cells four weeks after injection to the gingiva. Less than 1% of the nuclei were HNA-positive, suggesting that the main role of transplanted cells in MRONJ healing was via paracrine effect (Fig. 5e).

### EPC-CM recovered *in-vitro* wound healing via VEGF pathway

**Figure 6:**
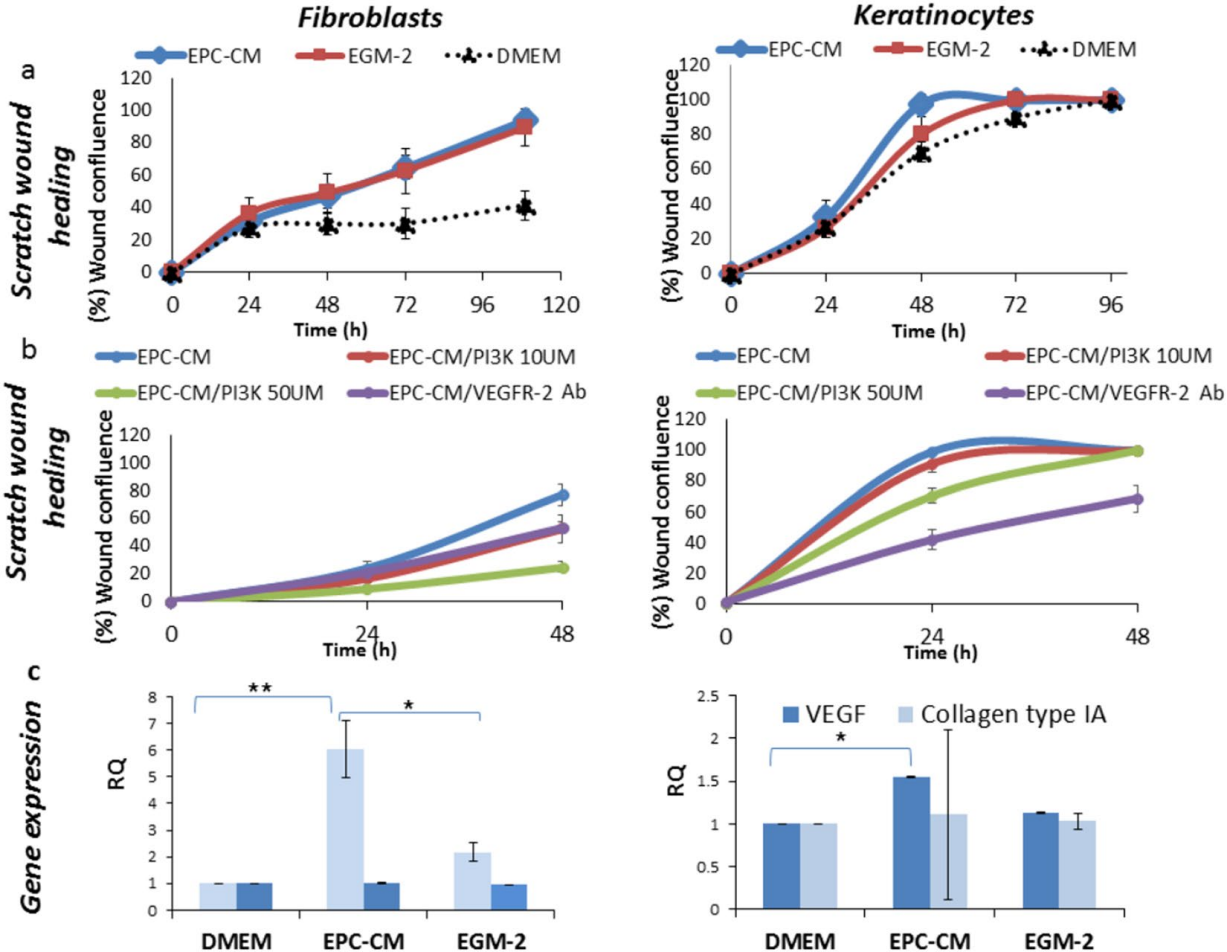
Effect of endothelial progenitor cells conditioned medium (EPC-CM) on gingival fibroblasts and keratinocytes scratch wound assay and associated genes. **(a)** Scratch wound assay: Cells were cultured in 10 µM zoledronic acid (ZOL) for 30 h. Immediately after scratch, the medium was replaced by fresh drug-free medium: Dulbecco’s modified Eagle medium (DMEM), EPC-CM, and endothelial growth medium without EPC secretome (EGM-2). Confluence of the wound was followed and measured automatically, *P* ≤ 0.038, EPC-CM vs. DMEM + drugs for gingival fibroblasts and keratinocytes. (**b**) Scratch wound healing assay with VEGF pathway blockage: Cells were cultured in 10 µM zoledronic acid (ZOL) for 30 h. Immediately after scratch, the medium was replaced by fresh drug-free medium: EPC-CM(+)/50 µM PI3K, EPC-CM(+)/10 µM PI3K or EPC-CM(+)/0.2 mg/ml VEGFR-2 antibody. (**c**) Wound healing associated genes: Cells were cultured in 10 µM zoledronic acid (ZOL) for 30 h, followed by 48 h in: DMEM, EPC-CM, or EGM-2. The mRNA of VEGFA and COL1A1 were quantified using RT-PCR. Relative quantification was calculated relative to HPRT and cell cultured in10 µM ZOL for 30 h followed by DMEM for 48 h. *P ≤ 0.05, **P ≤ 0.001. Experiments repeated tree times in triplicates.

The paracrine effect of EPC-CM on cell function was studied in a scratch wound healing assay. Gingival fibroblasts and keratinocytes were cultured with 10 µM ZOL for 30 h. Prior to scratch, medium was replaced by EPC-CM, EGM-2, or DMEM. For both cell types, treatment with EPC-CM, enhanced wound closure compared to cells exposed to ZOL and cultured in standard medium (DMEM) (*P* = 0.034, *P* = 0.038 for keratinocytes and GF, respectively). Culture in EGM-2 also accelerated wound closure compared to DMEM; but for keratinocytes greater improvement was found with EPC-CM (EPC-CM *vs*. EGM-2, *P* = 0.05; Fig. 6a).

In order to evaluate whether VEGF plays a role in cell recovery, concentrations of VEGF in the EPC-CM and DMEM were analyzed using an ELISA kit. VEGF concentration in EPC-CM was 126.012 ± 22.13 pg/ml and 7.8 ± 0.29 pg/ml in DMEM. Next, in VEGF neutralization experiment, a similar scratch wound healing assay was performed in the presence or absence of VEGFR-2 antibody. Gingival fibroblasts and keratinocytes were cultured with 10 µM ZOL for 30 h. Cells were seeded (2 × 10^4^ GF or 5 × 10^4^ keratinocytes) in ESSEN 96-well plates. When cells reached 95% confluence, a wound was made. Prior to scratch, medium was replaced by: EPC-CM (control) or EPC-CM(+)/0.2 mg/ml VEGFR-2 antibody (R&D Systems, MN, USA). Wound confluence of GF was significantly delayed by VEGFR-2 antibody (77.03% ± 7.72 vs. 53.36 ± 4.26, EPC-CM and VEGFR-2 antibody respectively, P = 0.00004). Likewise, keratinocytes wound healing was delayed by anti-VEGFR-2 (wound confluence was 41.81% ± 6.53 compared to 98.92% ± 0.91 in the EPC-CM, *P* = 0.01). In addition, VEGF pathway blocking was examined using PI3K (LY-294,002). Prior to scratch, medium was replaced by: EPC-CM(+)/50 µM PI3K (Sigma-Aldrich, MS, USA) or EPC-CM(+)/10 µM PI3K. For both cell types, blockage of VEGF pathway significantly delayed wound closure compared to EPC-CM (Fig. 6b) with a dose response effect. Wound confluence of GF decreased in 25% and 50% following incubation with10 or 50 µM PI3K compared to control (77.03% ± 7.72 in EPC-CM, 52.56% ± 9.72 in EPC-CM(+)/10 µM PI3K, 24.9% ± 3.79 in EPC-CM(+)/50 µM PI3K; P ≤ 0.0004). PI3K inhibitor also influenced keratinocytes function: wound confluence decreased by 28% using 50 µM PI3K (98.92% ± 0.91 in the EPC-CM vs. 70.23% ± 4.71, *P* = 0.007), without significant change for 10 µM PI3K.

## Discussion

The most accepted theory of MRONJ pathogenesis is impaired function of osteoclasts; it is therefore considered as a bone disorder. This study suggests an additional mechanism in MRONJ development that involves impaired wound healing of the oral mucosa caused by malvascularization and reduced fibroblast and keratinocyte function induced by ZOL and aggravated by DEX. Based on these findings, we examined the potential effects of proangiogenic cells to treat MRONJ, with promising results.

To gain insight into the pathogenesis of MRONJ, we used a previously established rat model^20,24^. As in human subjects, the combination of ZOL and DEX increased the prevalence of MRONJ compared to ZOL alone. Clinical and histological manifestations of MRONJ similar to humans were found only with ZOL and ZOL(+)/DEX(+)^25,26^. Therefore, we aimed to find a biological process that is uniquely disrupted by ZOL. The plasma concentration of ZOL in patients taking 2-4 mg of the drug, ranges between 1–10 µMZOL. Higher levels in the bone are expected due to accumulation and release of the drug from this tissue^27,28^. Gingival fibroblasts and keratinocytes cultured in 10 µMZOL or ZOL(+)/DEX(+) demonstrated significant impaired wound healing that was not observed in DEX. Previous studies showed reduced fibroblast and keratinocyte viability and metabolic activity with low doses of ZOL and up-regulation of apoptosis associated genes^11,29^. In contrast, DEX increased human fibroblast proliferation^30,31^.

In the *in-vivo* model, ZOL, DEX, and their combination reduced vascularity of the oral soft tissue, without an additive effect between ZOL and DEX. Suggested mechanisms of reduced vascularity by ZOL include: reduced HUVEC proliferation, migration and capillary-like formation^32,33^; reduced differentiation of MSCs to endothelial cells^34^; and decreased VEGF receptor binding on endothelial cells^35^. Reduction in tumor micro-vessels was also found in DEX-treated cancer patients^36,37^. Despite the anti-angiogenic effect of DEX, MRONJ did not develop, suggesting that impaired vascularity is not the primary cause of it.

Serum VEGF was decreased in ZOL(+)/DEX(+), ZOL, and DEX in this MRONJ rat model. Reduced serum VEGF levels were found after a single ZOL infusion in cancer patients^8^ as well following DEX treatment^38^. VEGF in the oral mucosa was dominant in the epithelium and reduced only in the ZOL and ZOL(+)/DEX(+) groups (not in DEX) suggesting that decreased tissue-VEGF may play a greater role than decreased serum-VEGF in MRONJ. VEGF stimulates epithelialization and collagen deposition, both imperative in wound healing^39,40^. Indeed, blockage of VEGF pathway significantly delayed scratch wound healing by GF and keratinocytes. VEGF plays a role in bone remodeling via induction of osteoclast differentiation from monocytes and increases RANKL expression by fibroblasts, which promotes osteoclasts differentiation^41^. Therefore, a negative effect of VEGF reduction on soft and hard tissue healing is relevant to MRONJ pathogenesis and can explain disease occurrence exclusively in the ZOL, but not in the DEX groups, as well as in patients taking anti-VEGF or BPs.

The novelty of using EPCs in MRONJ treatment is based on their significant role in angiogenesis^15^, improved bone repair^17,42–44^, and simple isolation from the patient’s blood. In this study, rats were treated with human cells, without clinical or histological evidence of an inflammation possibly due to immunosuppression of the rats by DEX^45^. MRONJ healing occurred in rats treated with EPCs along with increased serum and tissue VEGF, with less favorable results found in the MSC and medium groups. Since MSC also elevated serum and tissue VEGF, the differences between EPCs and MSCs can be attributed to the stronger potency of EPCs in angiogenesis and vasculogenesis; secretion of multiple proteins, including stromal derived factor -1 (SDF-1), which stimulates migration of osteoclasts^46,47^. Owing to minimal EPC engraftment, their paracrine effect is predominant. The EPC secretome consists of more than 300 molecules^48–50^ including VEGF. EPC-CM enhanced soft tissue wound healing via VEGF pathway and elevated COL1A1, and VEGFA in GF and keratinocytes.

In conclusion, the pathogenesis of MRONJ involves impaired function of keratinocytes and GF due to reduced COL1A1 and VEGFA, as well as malvascularization. A novel EPC treatment increased vascularization and improved cell function, thereby curing MRONJ. Future studies will focus on discovering the dominant molecules in EPC-CM responsible for modulating these healing processes.

## Supplementary information

Supplementary Dataset 1

## Data Availability

All data generated or analyzed during this study are included in this published article (and its Supplementary Information files).
