# Supplementary material for: Endothelial Progenitor Cells inhibit jaw osteonecrosis in a rat model: A major adverse effect of bisphosphonate therapy
Source: Sci Rep. 2019 Dec 11;9:18896. doi: 10.1038/s41598-019-55383-5 (PMC6906486; doi:10.1038/s41598-019-55383-5)
Supplement: Supplementary file 1 — Supplementary Dataset 1 [file 41598_2019_55383_MOESM1_ESM.docx]

**Supplementary- Scientific Reports**

**Endothelial Progenitor Cells Inhibit Jaw osteonecrosis in a rat model: A Major Adverse Effect of Bisphosphonate Therapy**

**Tal Tamari, PhD, Rina Elimelech, BDS, Gal Cohen, BSc, Talia Cohen, DVT, Ofri Doppelt, BSc, Lana Eskander-Hashoul, DMD, and Hadar Zigdon-Giladi, DMD, PhD**

**Dose response and time course effect of ZOL, DEX and ZOL(+)/DEX(+) on gingival fibroblasts proliferation**: to determine the concentration of ZOL and DEX for proliferation studies, cell were cultured in 0, 0.1 and 10 µM ZOL, DEX , ZOL(+)/DEX(+). XTT assay was performed according to the manufacture instructions at 24, 48 and 72h. 10µM ZOL or 10µM ZOL(+)/DEX(+) showed decreased proliferation between 24-72 h (concentration of 0.1µM ZOL or 0.1µM ZOL+DEX didn't effect cell proliferation). Cells culture in DEX demonstrated increased proliferation from 0 to 72 h.

**
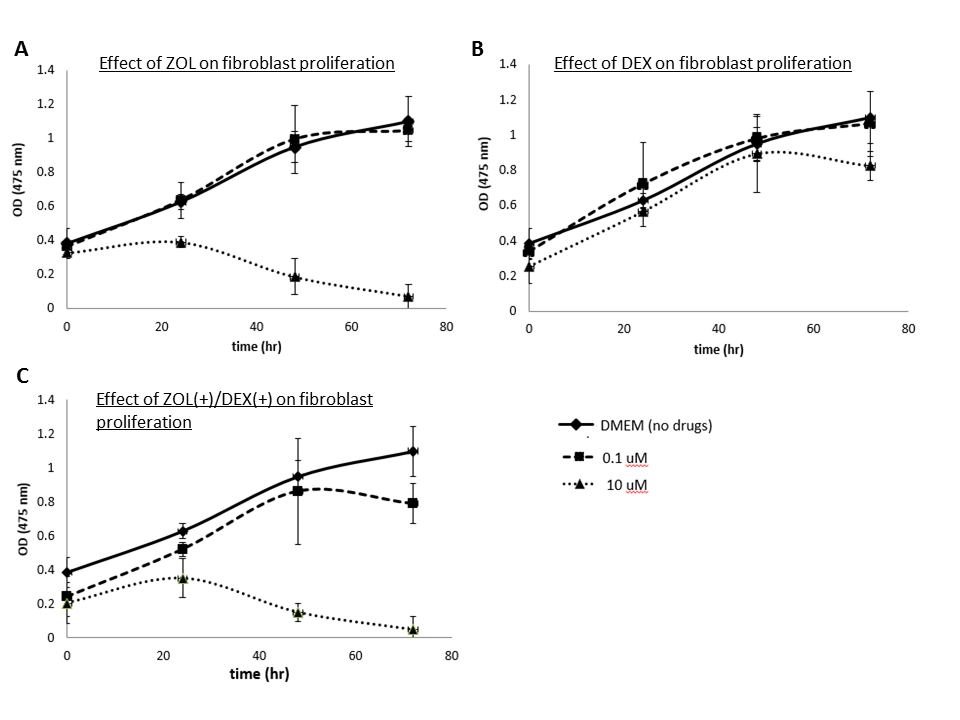
**

*Fig 1S- A dose response effect of zolendronic acid (ZOL) and dexamethasone (DEX) on gingival fibroblast (****A****) effect of ZOL on fibroblasts proliferation. (****B)*** *effect of DEX on fibroblasts proliferation (****C****) effect of ZOL(+)/DEX(+) on fibroblasts proliferation.*

**Dose response and time course effect of ZOL, DEX and ZOL(+)/DEX(+) on gingival fibroblasts scratch wound healing**: to determine the concentration of ZOL and DEX for scratch assays, cells were cultured until 95% confluence. After the scratch, the medium was replaced by 0.1 and 10 µM ZOL, DEX , ZOL(+)/DEX(+); DMEM without drugs served as control. Wound confluence was followed for 47h. Treatment with10µM ZOL or 10µM ZOL(+)/DEX(+) significantly delayed wound closure compared to DMEM (*P* = 0.004, *P* = 0.004, respectively). Conversely, treatment with 0.1 and 10 µM DEX or 0.1µM ZOL or 0.1µM ZOL(+)/DEX(+) showed slight and non-significant delay in wound closure compared to DMEM.


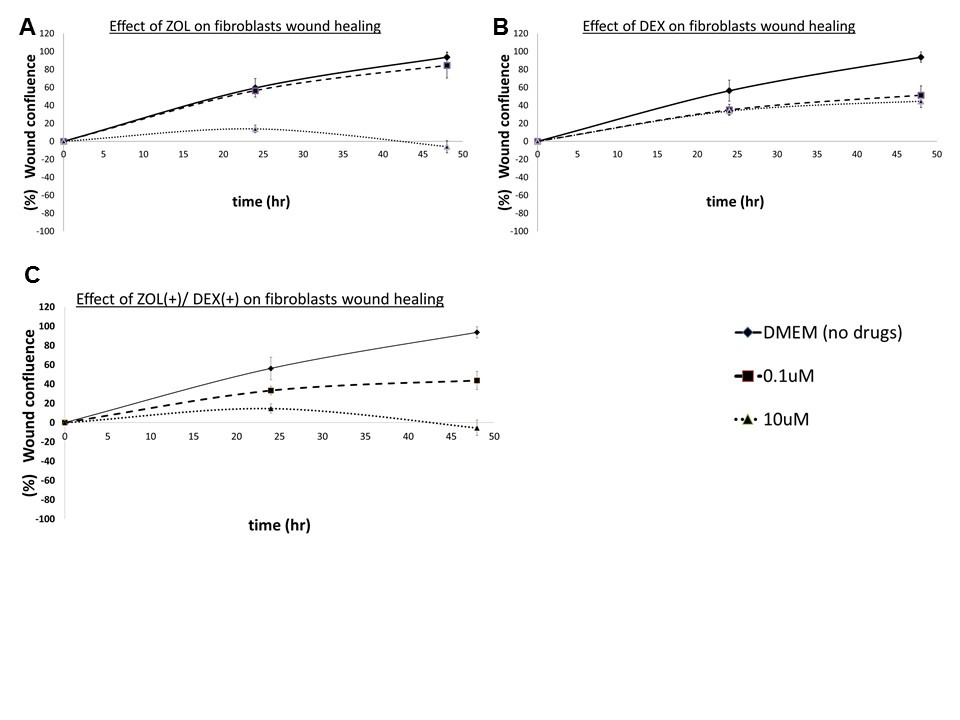


*Fig 2S- A dose response effect of zolendronic acid (ZOL) and dexamethasone (DEX) on gingival fibroblast wound healing(****A****) effect of ZOL on fibroblasts wound confluence. (****B)*** *effect of DEX on fibroblasts wound confluence (****C****) effect of ZOL(+)/DEX(+) on fibroblasts wound confluence.*

### **Expression of Receptor activator of nuclear factor kappa-Β ligand (RANKL) and Osteoprotegerin (OPG) genes in cells exposed to ZOL and or DEX**

To evaluate the effect of ZOL and DEX on RANKL and OPG genes expression levels, 2x10^5^ GF and keratinocytes were cultured for 30 h with DMEM+10 µM ZOL; 10 µM DEX; 10 µM ZOL(+)/DEX(+).Quantitative real-time PCR analysis (qPCR) was performed using real time PCR (Biometra Analytik, Jena, Germany) and syber green (Fast SYBR™ Green Master Mix, Applied Biosystems™, California, USA). All primers were supplied by Syntezza Bioscience (Jerusalem, Israel) RANKL forward: GCTTGAAGCTCAGCCTTTTG reverse: CGAAAGCAAATGTTGGCATA and OPG forward: TCAGAAAGGAAATGCAACACA, reverse: CCGTTTTATCCTCTCTACACT. The normalizing gene was HPRT forward: GACCAGTCAACAGGGGACAT, reverse: CCTGACCAAGGAAAGCAAAG. Data were analyzed with the relative Quantification-comparative CT method. RQ values were normalized to DMEM. Small but significant changes in RQ values were observed. A reduction in RANKL mRNA expression in GF and keratinocytes was found following exposure to ZOL compared to control. Whereas, Dexamethasone slightly increased RANKL expression in keratinocytes.

|  | RQ  (mean ± SD) | 10 µM ZOL | 10 µM DEX | 10 µM ZOL(+)/DEX(+) |
| --- | --- | --- | --- | --- |
| Gingival fibroblasts | RANKL | 0.71±0.21*** | 0.84±0.3 | 0.97±0.24 |
|  | OPG | 0.95±0.26 | 1.15±0.3 | 0.77±0.34 |
| Keratinocytes | RANKL | 0.76±0.1*** | 1.23±0.24* | 1.1±0.44 |
|  | OPG | 1±0.26 | 1.29±0.66 | 0.67±0.49 |

Table 1S- RQ levels of RANKL and OPG in fibroblasts and keratinocytes exposed to ZOL and or DEX.RQ were normalized relative to HPRT and DMEM. *** P<0.000 vs. DMEM; *P = 0.04 vs. DMEM.
